# Gender-specific effects of intrauterine growth restriction on the adipose tissue of adult rats: a proteomic approach

**DOI:** 10.1186/s12953-015-0088-z

**Published:** 2015-12-02

**Authors:** Adriana Pereira de Souza, Amanda Paula Pedroso, Regina Lúcia Harumi Watanabe, Ana Paula Segantine Dornellas, Valter Tadeu Boldarine, Helen Julie Laure, Claudia Maria Oller do Nascimento, Lila Missae Oyama, José Cesar Rosa, Eliane Beraldi Ribeiro

**Affiliations:** Departamento de Fisiologia, Universidade Federal de São Paulo, Rua Botucatu, 862 - 2 andar, Vila Clementino, São Paulo, SP 04023-062 Brazil; Centro de Química de Proteínas – Hemocentro, Universidade de São Paulo, Ribeirão Preto, Brazil

**Keywords:** Intrauterine growth restriction, Maternal undernutition, Adipose tissue, Proteome

## Abstract

**Background:**

Intrauterine growth restriction (IUGR) may program metabolic alterations affecting physiological functions and lead to diseases in later life. The adipose tissue is an important organ influencing energy homeostasis. The present study was aimed at exploring the consequences of IUGR on the retroperitoneal adipose tissue of adult male and female rats, using a proteomic approach.

**Methods and Results:**

Pregnant Wistar rats were fed with balanced chow, either *ad libitum* (control group) or restricted to 50 % of control intake (restricted group) during the whole gestation. The offspring were weaned to ad libitum chow and studied at 4 months of age. Retroperitoneal fat was analyzed by two-dimensional gel electrophoresis followed by mass spectrometry.

Both male and female restricted groups had low body weight at birth and at weaning but normal body weight at adulthood. The restricted males had normal fat pads weight and serum glucose levels, with a trend to hyperinsulinemia. The restricted females had increased fat pads weight with normal glucose and insulin levels.

The restricted males showed up-regulated levels of proteasome subunit α type 3, branched-chain-amino-acid aminotransferase, elongation 1- alpha 1, fatty acid synthase levels, cytosolic malate dehydrogenase and ATP synthase subunit alpha. These alterations point to increased proteolysis and lipogenesis rates and favoring of ATP generation.

The restricted females showed down-regulated levels of L-lactate dehydrogenase perilipin-1, mitochondrial branched-chain alpha-keto acid dehydrogenase E1, and transketolase. These findings suggest impairment of glycemic control, stimulation of lipolysis and inhibition of proteolysis, pentose phosphate pathway and lipogenesis rates.

In both genders, several proteins involved in oxidative stress and inflammation were affected, in a pattern compatible with impairment of these responses.

**Conclusions:**

The proteomic analysis of adipose tissue showed that, although IUGR affected pathways of substrate and energy metabolism in both males and females, important gender differences were evident. While IUGR males displayed alterations pointing to a predisposition to later development of obesity, the alterations observed in IUGR females pointed to a metabolic status of established obesity, in agreement with their increased fat pads mass.

**Electronic supplementary material:**

The online version of this article (doi:10.1186/s12953-015-0088-z) contains supplementary material, which is available to authorized users.

## Background

The concept of fetal programming describes that the exposition to adverse stimuli or insults, during critical phases of intrauterine development, may induce permanent changes in physiological functions and lead to adulthood diseases [1]. Increased risks of type 2 diabetes, insulin resistance, cardiovascular diseases and obesity have been associated with intrauterine growth restriction (IUGR) induced by undernutrition [2, 3]. These consequences have been shown to depend on the severity, duration and gestational period of the insult and also to be gender-dependent [4, 5]. Importantly, a mismatch between intrauterine and post-natal nutritional environment has been shown to be relevant for the expression of the programmed metabolic dysfunctions [6].

Previous reports have found that the adult offspring of IUGR rats displayed hyperphagia, obesity, hypertension, high serum leptin and insulin levels, increased hypothalamic density of leptin and serotonin receptors, and impairment of serotonin and insulin hypothalamic signaling [7–13]. Additionally, IUGR led to increased circulating levels of catecholamines in rats [14] and decreased levels of branched-chain amino acids in mice [15].

Lipogenesis and lipolysis are important physiologic pathways in the adipose tissue. Fatty acids for triacylglycerols synthesis may be taken up from the circulation or derive from *de novo* synthesis from glucose. Glucose degradation also yields glycerol 3-phosphate for fatty acids esterification and storage [16, 17]. Conversely, fatty acids and glycerol derived from triacylglycerols lipolysis may be released into the circulation. Those are hormone-controlled pathways. Lipolysis is inhibited by insulin and stimulated by cathecolamines and growth hormone. On the other hand, lipogenesis is stimulated by insulin while growth hormone is inhibitory [16–18]. Rat studies have indicated that IUGR due to maternal food restriction decreased adipose tissue lipolysis while it increased lipogenesis and/or adipogenesis, due to impairment of sympathetic activity [2].

The adipose tissue is also an endocrine organ whose secretions influence the onset of metabolic disorders [19]. Increased production of pro-inflammatory adipokynes in obesity plays a relevant role in the linking of adiposity, metabolic syndrome and cardiovascular diseases [20, 21]. Recent reports have shown that fetal leptin and adiponectin levels closely related to birth weight and IUGR has been shown to increase leptin but not adiponectin levels. Moreover, TNF-α levels have been found to be either normal or increased while IL-6 levels were either increased or decreases in IUGR [19, 22, 23].

Proteomic analysis allows the exam of hundreds of proteins in a sample and the identification of modification on their expression pattern in response to physiologic, pathologic and nutritional alterations, possibly leading to the identification and characterization of biological markers [24, 25]. Two-dimensional gel electrophoresis (2DE) followed by mass spectrometry remains an effective methodology in proteomics, especially as an initial approach [26, 27]. Recent proteomic studies have focused on the consequences of IUGR in tissues of animals and humans. Down-regulation of proteins related to oxidative phosphorylation has been found in the liver of both male and female IUGR rats [28]. In piglets, IUGR up-regulated subcutaneous adipose tissue levels of proteins related to glucose and fatty acid metabolism, lipid transport and apoptosis [29]. In humans, IUGR has been shown to increase serum levels of proteins related to signal transduction, blood coagulation and antioxidant response, while immune response proteins were down-regulated [30].

The above data indicate that IUGR may injure multiple aspects pertinent to adipocytes physiology that are relevant to the development of metabolic impairment and obesity in adulthood. Considering the above, the objective of this study was to further explore the consequences of IUGR in the adipose tissue of adult rats through proteomic approach.

## Results

### Body and white adipose tissue weight and blood and tissue parameters

Restricted male and female rats had low body weight at birth and at weaning but this difference was no longer observed at four months of age (Table 1). Food intake was similar between control and restricted animals, from weaning to 4 months of age (data not shown).

**Table 1:** Body and white adipose tissue weight of male and female control and restricted offspring

	Male		Female	
	Control (16)	Restricted (17)	Control (14)	Restricted (14)
BW at birth (g)	6.08 ± 0.11	4.84 ± 0.12***	5.69 ± 0.14	4.80 ± 0.13***
BW at weaning (g)	85.44 ± 2.32	76.05 ± 2.52**	79.83 ± 2.15	70.24 ± 2.68**
BW at 4-months (g)	390.5 ± 7.2	385.4 ± 10.6	234.9 ± 3.3	227.2 ± 4.2
BW gain (g)	263.6 ± 8.4	271.2 ± 10.8	121.2 ± 4.8	127.2 ± 5.5
Retroperitoneal (g/100 g bw)	1.15 ± 0.11	1.24 ± 0.11	0.80 ± 0.06	0.95 ± 0.06
Mesenteric (g/100 g bw)	0.91 ± 0.07	0.98 ± 0.07	1.01 ± 0.05	1.19 ± 0.04**
Gonadal	1.31 ± 0.11	1.50 ± 0.08	2.46 ± 0.19	3.0 ± 0.17*
Total weight (g/100 g bw)	3.37 ± 0.28	3.72 ± 0.23	4.27 ± 0.28	5.22 ± 0.22*

Data are means ± SEM; (number of animals)

*BW* body weight, *g/100 g bw* grams/100 g of body weight, *Total weight* sum of retroperitoneal, mesenteric and gonaldal fat pads

*p < 0.01 vs. control

**p < 0.01 vs. control

***p < 0.001 vs. control

White adipose tissues weight were similar between control and restricted males. The female restricted rats showed increased weight of mesenteric and gonadal white adipose tissue and the sum of the three fat pads was higher than that of the control females (Table 1).

Serum glucose, adiponectin, corticosterone and triglycerides were similar between the groups of male rats. Serum insulin levels showed a tendency to increase in the restricted males (p = 0.083). Serum and tissue cytokines levels were similar between the male groups (Table 2).

**Table 2:** Blood and retroperitoneal adipose tissue parameters of adult male and female control and restricted offspring

	Male		Female	
	Control	Restricted	Control	Restricted
Serum glucose (mg/dl)	100.2 ± 5.2 (18)	92.4 ± 3.1 (9)	95.2 ± 3.9 (13)	100.0 ± 5.2 (9)
Serum Insulin (ng/ml)	0.50 ± 0.07 (8)	0.79 ± 0.13 (9)	0.35 ± 0.06 (9)	0.34 ± 0.12 (9)
Serum adiponectin (μg/ml)	12.3 ± 1.4 (9)	11.3 ± 1.1 (9)	17.45 ± 1.81 (9)	18.47 ± 2.27 (9)
Serum corticosterone (ng/ml)	94.2 ± 7.9 (9)	97.0 ± 8.8 (8)	63.58 ± 9.54 (9)	71.06 ± 5.58 (9)
Serum triglycerides (mg/dl)	55.1 ± 4.3 (18)	68.6 ± 9.3 (9)	37.2 ± 3.1 (14)	38.7 ± 6.0 (8)
Serum TNF-α (pg/ml)	7.6 ± 1.7 (8)	18.2 ± 6.4 (8)	20.07 ± 1.8 (8)	14.78 ± 4.3 (9)
Serum IL-1β (ng/ml)	0.10 ± 0.04 (6)	0.06 ± 0.01 (6)	0.14 ± 0.04 (9)	0.04 ± 0.01*(8)
Tissue TNF- α (pg/ml)	49.3 ± 4.5 (8)	55.0 ± 3.2 (8)	63.93 ± 4.9 (8)	71.55 ± 4.3 (8)
Tissue IL-6 (pg/ml)	107.3 ± 7.0 (8)	106.6 ± 6.9 (8)	126.24 ± 3.8 (8)	134.13 ± 6.9 (8)
Tissue IL-10 (pg/ml)	187.6 ± 19.2 (8)	225.0 ± 28.7 (8)	275.89 ± 19.5 (8)	311.09 ± 19.5 (8)

Data are means ± SEM. (number of animals)

*p < 0.05 vs control

For female rats, no differences were found in serum glucose, insulin, corticosterone, adiponectin and triglycerides between the control and restricted groups. The restricted females showed low serum IL-1β (Table 2).

### Proteomic analysis

**Fig. 1:**
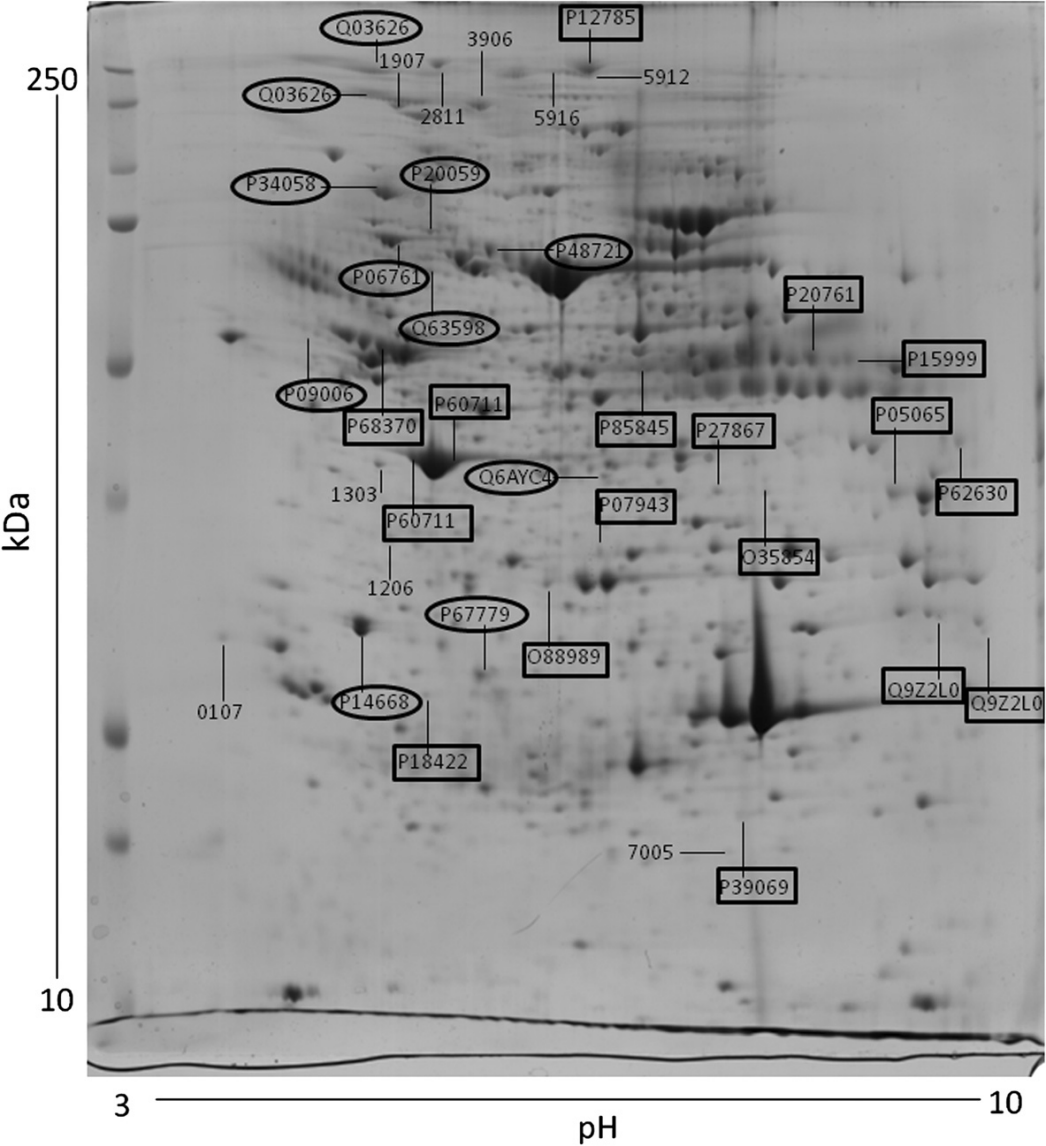
Representative image of a 2DE gel of a control male retroperitoneal adipose tissue depicting the proteins significantly affected by IUGR. The numbers indicate the protein acession number. Numbers in squares indicate the over-expressed identified proteins. Numbers in circles indicate the under-expressed identified proteins

The 2DE gels of retroperitoneal adipose tissue showed 425 ± 2.9 spots in control males (*N* = 8) and 417 ± 4.5 spots in restricted males (*N* = 8). Of these, 37 spots showed significant density changes, with 15 spots under- and 22 over-expressed. Spots optic densities are shown in Additional file 1: Table S1. The significantly affected spots were analyzed by mass spectrometry for proteins identification. Figure 1 shows a representative image of a 2DE gel of a control male with indication of the spots significantly affected by IUGR.

The MS analysis identified 11 of the 15 under-expressed proteins and 17 of the 22 over-expressed proteins. One down-regulated protein (Murinoglobulin-1) and 2 up-regulated proteins (Actin, cytoplasmic I and Voltage-dependent anion-selective channel protein 1) were identified in 2 adjacent spots. Table 3 shows the Swiss-Prot Accession Numbers (available at http://www.expasy.ch/sprot), full protein names, theoretical molecular weight (MW) and isoelectric point as well as the mass spectrometry data of the identified proteins having statistically significant Mascot score results (*p* < 0.05) in males. Additional file 2: Table S2 shows gene names and biological processes of the proteins significantly up-regulated and down-regulated proteins, as assessed by Panther software. Metabolic process was the most common biological process class for both the down-regulated (7 out of 10) and the up-regulated (9 out of 15) proteins. The metabolic processes included lipidic, amino acid and carbohydrate metabolism (Table 4).

**Table 3:** Identified proteins with significant expression alteration between control and restricted males

Accession Number	Protein Name	Matched Peptides	Score	Coverage (%)	Fold Change (R/C)	MW (Da)/ pI
*Down-regulated Proteins*						
P06761	78 kDa glucose-regulated protein	6	334	11	0.60	71476/5.07
P14668	Annexin A5	6	338	18	0.39	35780/4.93
P34058	Heat shock protein HSP 90 β	9	245	12	0.68	83577/4.97
P20059	Hemopexin	4	66	6	0.55	52072/7.58
Q6AYC4	Macrophage-capping protein	1	50	3	0.63	39065/6.11
Q03626	Murinoglobulin-1	3	66	3	0.47	166614/5.68
Q03626	Murinoglobulin-1	2	81	1	0.24	166614/5.68
Q63598	Plastin-3	1	46	1	0.22	71157/5.32
P67779	Prohibitin	4	196	17	0.58	28860/5.57
P09006	Serine protease inhibitor A3N	3	109	6	0.37	46796/5.33
P48721	Stress-70 protein, mitochondrial	3	66	6	0.52	74102/5.97
*Up-regulated Proteins*						
P60711	Actin, cytoplamic I	6	191	21	1.77	42058/5.29
P60711	Actin, cytoplamic I	4	244	15	1.83	42058/5.29
P39069	Adenylate kinase izoenzyme 1	2	30	12	1.96	21686/7.66
P07943	Aldose reductase	3	94	10	2.05	36238/6.26
P15999	ATP synthase subunit alpha, mitochondrial	1	57	1	1.94	59833/9.22
O35854	Branched-chain-amino-acid aminotransferase. mitochondrial	4	52	9	1.67	44827/8.46
P62630	Elongation factor 1-alpha 1	2	52	4	1.79	50430/9.10
P85845	Fascin	3	39	7	2.27	55211/5.96
P12785	Fatty acid synthase	5	151	2	2.40	275146/5.96
P05065	Fructose-biphosphate aldolase A	7	245	17	1.49	39791/8.31
P20761	Ig gamma-2B chain C region	2	38	4	2.07	37112/7.70
O88989	Malate dehydrogenase, cytoplasmic	3	81	11	2.44	36634/6.16
P18422	Proteasome subunit α-type 3	4	86	15	1.93	28633/5.29
P27867	Sorbitol dehydrogenase	2	94	7	2.05	38790/7.14
P68370	Tubulin α-1A chain	3	121	9	2.37	50800/4.94
Q9Z2L0	Voltage-dependent anion-selective channel protein 1	2	60	8	2.16	30853/8.62
Q9Z2L0	Voltage-dependent anion-selective channel protein 1	1	42	4	4.94	30853/8.62

Accession number, protein name, number of matched peptides, proein score, percentage coverage, fold change (restricted/control) and theoretical molecular mass (Da) and pI of identified proteins

**Table 4:** Biological process classification of identified proteins of male rats

Protein name	Metabolic process
Down-regulated in restricted males	
Serine protease inhibitor A3N	Proteolysis
Murinoglubulin-1	
Hemopexin	
78 kDa glucose-regulated protein	Protein folding
Heat shock protein HSP 90-beta	
Prohibitin	DNA replication
Annexin A5	Lipidic
Up-regulated in restricted males	
Branched-chain-amino-acid aminotransferase, mitochondrial	Amino acid
Proteasome subunit α type-3	Proteolysis
Fatty acid synthase	Lipidic
Sorbitol dehydrogenase	Carbohydrate
Malate dehydrogenase, cytoplasmic	Carbohydrate, tricarboxilic acid cycle (TCA)
ATP synthase subunit alpha, mitochondrial	Respiratory chain
Adenylate kinase isoenzyme 1	Nucleotide
Elongation factor 1-alpha 1	Translation
Aldose reductase	Transport

**Fig. 2:**
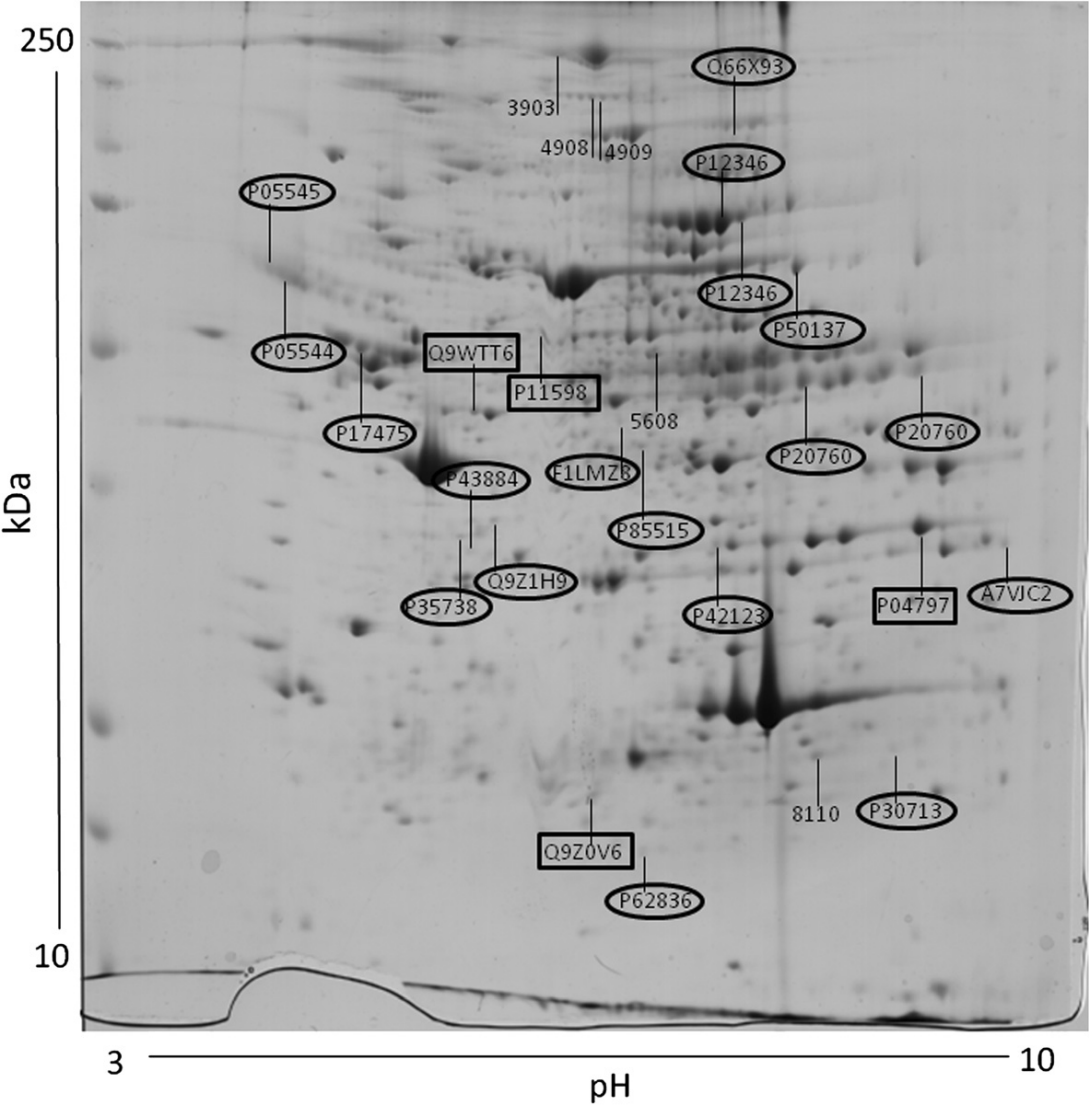
Representative image of a 2DE gel of a control female retroperitoneal adipose tissue depicting the proteins significantly affected by IUGR. The numbers indicate the protein acession number. Numbers in squares indicate the over-expressed identified proteins. Numbers in circles indicate the under-expressed identified proteins

The 2DE gels of females had 404 ± 3.6 spots in the controls (*N* = 6) and 397 ± 3.9 spots in the restricted ones (*N* = 6). Of these, 27 spots showed significant density changes, with 20 spots under- and 7 over-expressed. Spots optic densities are shown in Additional file 1: Table S1. The significantly affected spots were analyzed by mass spectrometry for proteins identification. Figure 2 shows a representative image of a 2DE gel of a control female with indication of the spots significantly affected by IUGR.

The MS analysis identified 18 of the 20 down-regulated proteins and 4 of the 7 up-regulated proteins. Two down-regulated proteins (Serotransferrin and Ig gamma 2-A chain C) were identified in 2 adjacent spots. Female proteins data of significant Mascot score results (*p* < 0.05) are shown in Table 5. Additional file 3: Table S3 shows gene names and biological processes of the significantly up-regulated and down-regulated proteins in females. Metabolic process was the most common biological process class for the down-regulated (13 out of 16) and the up-regulated (4 out of 4) proteins. The metabolic processes included lipidic, amino acid and carbohydrate metabolism (Table 6).

**Table 5:** Identified proteins with significant expression alteration between control and restricted females

Accession number	Protein name	Matched peptides	Score	Coverage (%)	Fold change (R/C)	MW (Da)/ pI
*Down-regulated proteins*						
F1LMZ8	26S proteasome non-ATPase regulatory subunit 11	2	89	4	0.44	47724/6.08
P35738	2-oxoisovalerate dehydrogenase subunit β. mitochondrial	1	45	4	0.46	43550/6.41
P30713	Glutathione S-transferase theta-2	1	47	5	0.38	27596/7.75
A7VJC2	Heterogeneous nuclear ribonucleoproteins A2/B1	4	96	13	0.35	37513/8.97
P20760	Ig gamma 2-A chain C	3	79	9	0.53	35685/7.72
	Ig gamma 2-A chain C	4	109	13	0.23	
P42123	L-lactate dehydrogenase B	1	30	2	0.57	36879/5.70
P43884	Perilipin 1	2	74	5	0.41	55986/6.37
Q9Z1H9	Protein kinase C delta binding protein	5	119	18	0.33	27894/5.79
P62836	Ras-related protein Rap-1A	1	39	5	0.32	21322/6.38
P05545	Serine protease inhibitor A3K	3	119	10	0.45	46764/5.31
P05544	Serine protease inhibitor A3L	1	46	2	0.29	46442/5.48
P12346	Serotransferrin	6	133	8	0.38	78550/7.14
P12346	Serotransferrin	2	110	3	0.31	78550/7.14
Q66X93	Staphylococcal nuclease domain-containing protein 1	3	38	4	0.39	103585/6.76
P50137	Transketolase	3	115	9	0.45	68355/7.23
P17475	α-1 antiproteinase	6	255	14	0.50	46281/5.70
P85515	α-centractin	2	57	8	0.63	42703/6.19
*Up-regulated proteins*						
P04797	Glyceraldehyde-3-phosphate dehydrogenase	1	34	4	2.49	36095/8.14
Q9WTT6	Guanine deaminase	6	312	16	1.54	51564/5.56
P11598	Protein disulfide-isomerase A3	5	117	119	3.20	57052/5.88
Q9Z0V6	Thioredoxin-dependent peroxide reductase. mitochondrial	2	78	9	2.09	28567/7.14

Accession number, protein name, number of matched peptides, proein score, percentage coverage, fold change (restricted/control) and theoretical molecular mass (Da) and pI of identified proteins

**Table 6:** Biological process classification of identified proteins of female rats

Protein name	Metabolic process
Down-regulated in restricted females	
Serine protease inhibitor A3K	Proteolysis
Serine protease inhibitor A3L	
26S proteasome non-ATPase regulatory subunit 11	
Alpha-1-antiproteinase	
Transketolase	Carbohydrate, Amino acid, Lipidic
2-oxoisovalerate dehydrogenase subunit beta, mitochondrial	
Perilipin-1	Lipidic
Glutathione S-transferase theta-2	Protein
L-lactate dehydrogenase B chain	Glycolysis, TCA
Heterogeneous nuclear ribonucleoproteins A2/B1	Nucleotide
Protein kinase C delta-binding protein	Transcription
Staphylococcal nuclease domain-containing protein 1	
Ras-related protein Rap-1A	
Up-regulated in restricted females	
Guanine deaminase	Purine
Protein disulfide-isomerase A3	Protein folding
Glyceraldehyde-3-phosphate dehydrogenase	Glycolysis
Thioredoxin-dependent peroxide reductase, mitochondrial	

Classification of proteins in metabolic process

In both males and females, some proteins were identified in 2 adjacent spots, what may possibly be attributed to the existence of either different isoforms or post-translational modifications of the protein.

#### Western blot analysis

**Fig. 3:**
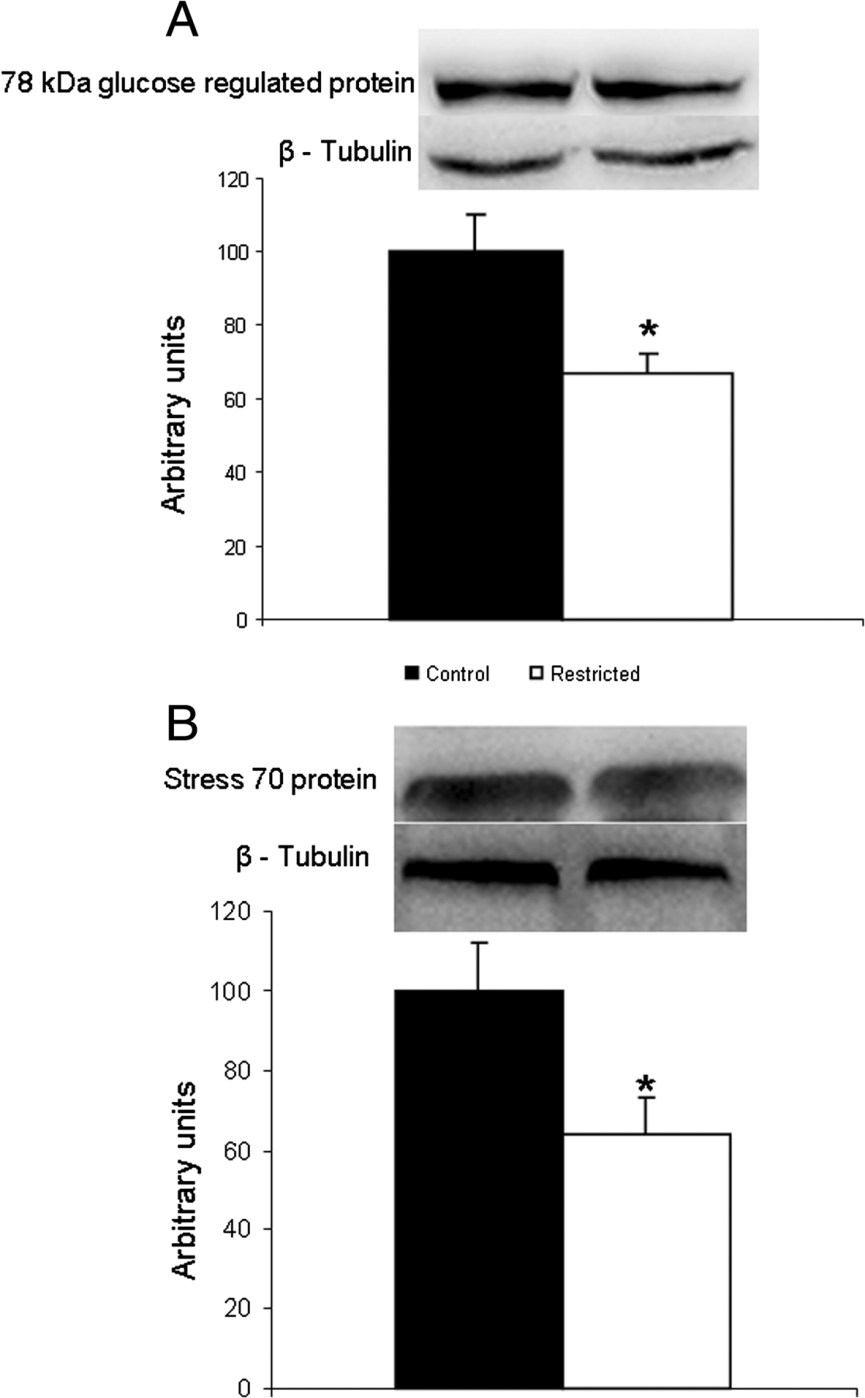
Western Blot analysis of 78 kDa glucose-regulated protein and Stress-70 protein. **a** 78 kDa glucose-regulated protein. *N* = 8 control; *N* = 9 restricted. **b** Stress 70 protein, mitochondrial. *N* = 10 controls; *N* = 12 restricted. *p < 0.05 vs. control

A sub-set of selected proteins was analyzed by Western blotting to confirm the proteome results. Corroborating the male proteome result of a 40 % decrease in expression of 78 kDa glucose-regulated protein in the restricted males, the western blot analysis showed a 33 % decrease. The mitochondrial stress-70 protein showed a 48 % decrease in the proteome experiment and a 36 % decrease in the western blot experiment (Fig. 3).

**Fig. 4:**
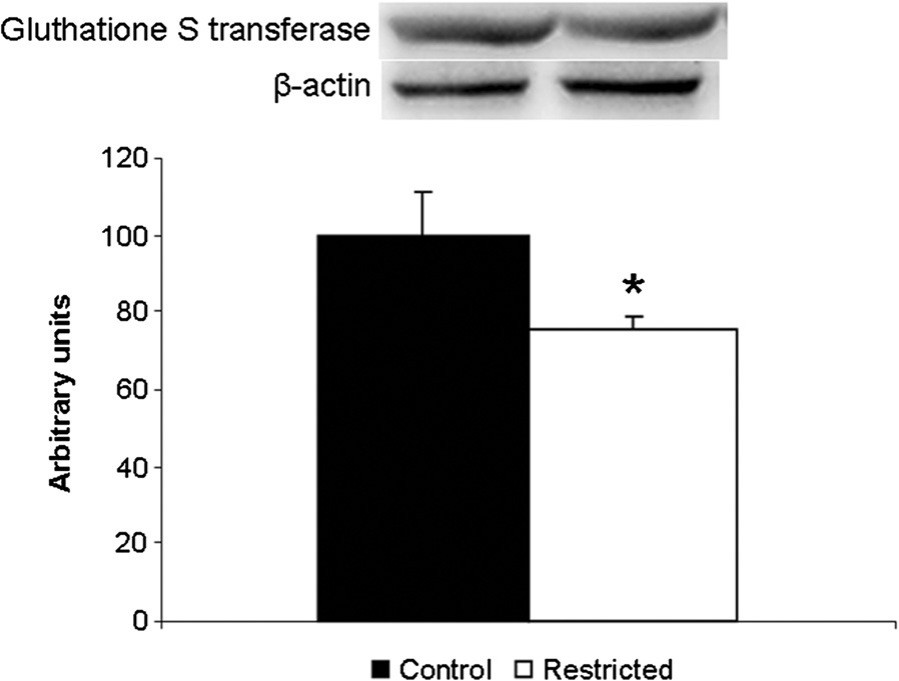
Western Blot analysis of glutathione S-transferase theta 2. *N* = 8 controls; *N* = 9 restricted. * = *p* < 0.05 vs. control

In the females, the proteome results showed a 68 % decrease of Glutathione S-transferase theta-2 in the restricted group while the western blot analysis showed a 25 % decrease (Fig. 4).

## Discussion

Male and female rats submitted to intrauterine growth restriction had normal food intake and body weight as adults, indicating catch-up growth, an adaptive mechanism against obesity in adult life [31, 32].

The restricted females, unlike the restricted males, showed increased fat pads weight, without overt peripheral insulin resistance, in accordance with a previous work from our laboratory [11]. This observation agrees with other reports showing the gender-dependency of the late consequences of rat maternal nutritional restriction [4, 5, 7–9]. In humans, early intrauterine undernutrition increased body mass index in 50 year-old women but not men [33].

The proteomic analysis of the adipose tissue of the males showed that IUGR caused alterations in the protein levels of 28 identified proteins. Levels of fatty acid synthase, enzyme of the *de novo* lipogenesis pathway [16], were increased by IUGR, in agreement with other reports [34, 35]. A study comparing lean and obese subjects found that increased fatty acid synthase gene expression was linked to visceral fat accumulation [36].

Prohibitin levels were down-regulated in the restricted males. This protein has been shown to attenuate insulin-stimulated oxidation of glucose and fatty acids in adipose tissue [37]. Over-expression of prohibitin in mice adipose tissue increased fat pads [38]. In contrast, knockdown of prohibitin in 3 T3-L1 pre-adipocytes increased oxidative stress due to impairment of mitochondrial function [39].

These protein expression alterations found in the restricted males, one favoring and the other counteracting lipid accumulation, may represent a pre-obese condition. Although the restricted males did not have augmented fat pads mass, they did show a tendency to hyperinsulinemia, suggesting that an increase in lipid synthesis could lead to obesity later in life.

The increased levels of proteasome subunit α type-3 suggest that IUGR caused stimulation of proteolysis in males. The adipose tissue has been shown to be an important site of proteolysis and to contribute to the circulating amino acids pool [40, 41]. Obese women showed a decreased rate of amino acids release from the tissue, in response to fasting [42].

In the adipose tissue of obese humans, levels of mitochondrial branched-chain-amino-acid aminotransferase were reportedly decreased from lean levels in the metabolically unhealthy but not in the healthy subset of obese subjects [43]. Here, tissue levels of mitochondrial branched-chain-amino-acid aminotransferase were increased in the restricted males, indicating that their metabolism was not affected at the same extent as that seen in unhealthy obesity.

IUGR up-regulated the levels of elongation factor 1-alpha 1, a GTPase that delivers aminoacyl–tRNAs to ribosomes during protein translation [44, 45]. This protein has been shown to interact with nascent proteins ubiquitinated during translation, facilitating their delivery to proteasome [46] and to be associated with stimulation of cell proliferation in cancer cells [47]. In kidneys of streptozotocin diabetic rats, increased expression of elongation factor-1A has been related to hypertrophy of the adipose organ and to diabetes-associated oxidative stress [48].

Obesity has recently been associated with increased levels of several amino acids in the visceral adipose tissue of humans [49]. Moreover, metabolomic analysis showed increased levels of phenylalanine, tryptophan and glutamate in the umbilical vein blood of IUGR neonates [50]. The increased levels of proteins related to proteolysis stimulation, as observed in the present study, may increase adipose tissue levels of amino acids. These may be converted to intermediates of the tricarboxylic acid (TCA) cycle. It is important to point out that, once entering the TCA cycle, these amino acids could be directed to either complete oxidation or generation of citrate [51], an important precursor for *de novo* lipogenesis. It is thus reasonable to suggest that proteolysis stimulation in the restricted males may provide amino acids for metabolic reactions in the tissue, rather than for release. A recent review has indicated that impairment of TCA cycle metabolites by IUGR could be an important biomarker of this condition [52].

Cytosolic malate dehydrogenase levels were up-regulated in the restricted males. This enzyme is active in the malate/aspartate shuttle, where it catalyzes the reduction of oxaloacetate to malate, using NADH. Malate enters mitochondria and is oxidized to oxaloacetate by mitochondrial malate dehydrogenase, with production of NADH. This shuttle not only channels the NADH produced during glycolysis to ATP production but also maintains the cytosolic NAD+/NADH ratio, essential for the oxidative metabolism of glucose [53–55]. Increased levels of mitochondrial malate dehydrogenase have been reported in pancreatic islets of adult rats with IUGR. However, ATP levels were not altered, which was attributed to the concomitant decrease of ATP synthase subunit 6 levels [55]. In the present study, ATP synthase subunit alpha was up-regulated, indicating that ATP production could be increased.

Some proteins down-regulated by IUGR in males are related to inflammation and cellular stress. Murinoglobulin-1 is a serino-protease inhibitor [56] that plays a protective role in the inflammatory response. Hemopexin is a positive acute-phase reactant that plays a protective role in lipid peroxidation through its heme binding effect [57], its levels being negatively associated with the severity of chronic sepsis [58]. In diet-induced obese mice, up-regulation of serum hemopexin levels has been suggested to represent a dysfunctional response in this chronic inflammatory condition [59].

The 78 kDa glucose-regulated protein is related to proper protein folding, protecting the cell from endoplasmic reticulum stress [60, 61], which has been described to link obesity to insulin resistance [61]. Obese mice overexpressing 78 kDa glucose-regulated protein in pancreas were protected against endoplasmic reticulum stress and had improvement of insulin sensitivity [62]. Heat shock protein HSP 90-beta is an important chaperone whose levels reportedly increase in obese humans, playing a role in mitigating the inflammatory stress present in obesity [63, 64]. Taken together, these protein alterations indicate that the restricted males presented impairment of anti-inflammatory reactions in the adipose tissue.

Overall, the results found in the male rats indicate that, even though the restricted males did not have augmented fat pads or glucose intolerance, the alterations in adipose tissue metabolism point to a tendency to develop obesity.

In the females, IUGR affected the glycolysis/gluconeogenesis pathway. L-lactate dehydrogenase B was down-regulated in the restricted females, indicating low production of lactate from pyruvate. Due to its low blood supply, the adipose tissue produces considerable amounts of lactate, which can serve either as precursor to energy production or fatty acid synthesis [65, 66] or be released to the systemic circulation [67], even in normoxia conditions [68]. Adipose tissue lactate production has been shown to correlate with lactate dehydrogenase activity, both under normal and cafeteria diet feeding, and suggested to contribute to glycemic control, through consumption of excess circulating glucose [69].

Glyceraldehyde-3-phosphate dehydrogenase, the enzyme catalyzing the reversible conversion of glyceraldehyde-3-phosphate to 1,3 bisphosphoglycerate and NADH, was up-regulated in the restricted females. Stimulation of glyceraldehyde-3-phosphate dehydrogenase has been reported in pre-obese, normoinsulinemic, Zucker rats [70]. Maternal peri-conceptional overnutrition, but not food restriction, increased fat mass of postnatal female lambs and glyceraldehyde-3-phosphate dehydrogenase gene expression correlated positively with perirenal fat amount [71].

Transketolase was down-regulated in the restricted females. This enzyme catalyzes the formation of glyceraldehyde-3-phospate in the non-oxidative branch of the pentose phosphate pathway, in which ribose is re-converted to glucose. Moreover, the pentose phosphate pathway generates NADPH for lipid synthesis. In obese individuals, decreased activity of the lipogenic pathway, with down-regulation of transketolase, has been interpreted as a mechanism aimed at reducing the growth of adipose tissue [72].

Reduced levels of mitochondrial branched-chain-amino-acid aminotransferase and mitochondrial 2-oxoisovalerate dehydrogenase (also known as branched-chain alpha-keto acid dehydrogenase E1), enzymes participating in the pathway of degradation of branched-chain amino acids, were found in the subcutaneous adipose tissue of unhealthy obese humans but not in the healthy obese subset [43]. Here, the latter enzyme was down-regulated in the restricted females. It is possible to suggest that the metabolism of branched-chain-amino-acids in the adipose tissue of the restricted females resembled that found in obesity associated with metabolic derangements. This contrasts with the result in the restricted males, in which mitochondrial branched-chain-amino-acid aminotransferase was increased.

The restricted females also showed down-regulation of perilipin-1, an enzyme active in lipid droplet formation [73] and inversely correlated with adipocyte size and basal lipolysis [74]. Perilipin gene suppression increased basal lipolysis and prevented high-fat diet obesity in mice [75].

Glutathione S-transferase theta-2 was down-regulated in the restricted females. This protein is part of the antioxidant enzymes family, which catalyzes the conjugation of glutathione to a wide variety of compounds. Decreased glutathione or glutathione-S transferase levels have been linked to diabetes, due to its role in antioxidant pathways [76, 77]. On the other hand, high levels of glutathione-S transferase P in obese subjects activated inflammatory pathways and endoplasmic reticulum stress [78].

Other proteins related to antioxidant pathways were up-regulated by IUGR in the females. Protein disulfide-isomerase A3 is a thiol-disulfide oxidoreductase present in the endoplasmic reticulum and it catalyzes the formation, breakdown and rearrangement of disulfide bonds [79]. Increased levels of protein disulfide-isomerase A3 in the adipose tissue of obese subjects have been suggested to activate inflammatory pathway and endoplasmic reticulum stress [79]. Mitochondrial thioredoxin-dependent peroxide reductase regulates H_2_O_2_ levels, protecting the cell from the toxicity resulting from its accumulation [80], and depletion of this protein accelerated apoptosis [81].

A protein down-regulated in the restricted females, α-1-antiproteinase, also known as serpin A1 and α1-antitrypsin, is a serine protease inhibitor with anti-inflammatory effects. It caused inhibition of lipopolysaccharide-mediated activation of in vitro human monocytes [82] and inhibited lung neutrophil chemotaxis [83–85]. Inhalation of α-1-antiproteinase decreased protein levels of IL-1β and IL-8 [86] while addition of purified plasma α-1-antiproteinase to pancreatic β-cells in vitro inhibited cytokine-induced apoptosis [87]. Alpha-1-antiproteinase gene therapy prevented the development of type 1 diabetes in non-obese mice [88]. Decreased levels of α-1-antiproteinase was reported by proteomic analysis of adipose tissue of women with gestational diabetes mellitus [89] impair the protection against inflammation and oxidative stress, compensatory mechanisms were recruited in the restricted females.

## Conclusions

In the restricted males, the high levels of proteasome subunit α type 3, branched-chain-amino-acid aminotransferase and elongation 1- alpha 1 indicate increased proteolysis rate in the adipose tissue. High tissue levels of amino acids could generate lipogenesis precursors, a suggestion supported by the high levels of fatty acid synthase. The increased levels of cytosolic malate dehydrogenase and ATP synthase subunit alpha may favor ATP production. These results indicate that, in the restricted males, the alterations in protein expression induced by IUGR pointed to a metabolic status favoring the development of obesity.

**Fig. 5:**
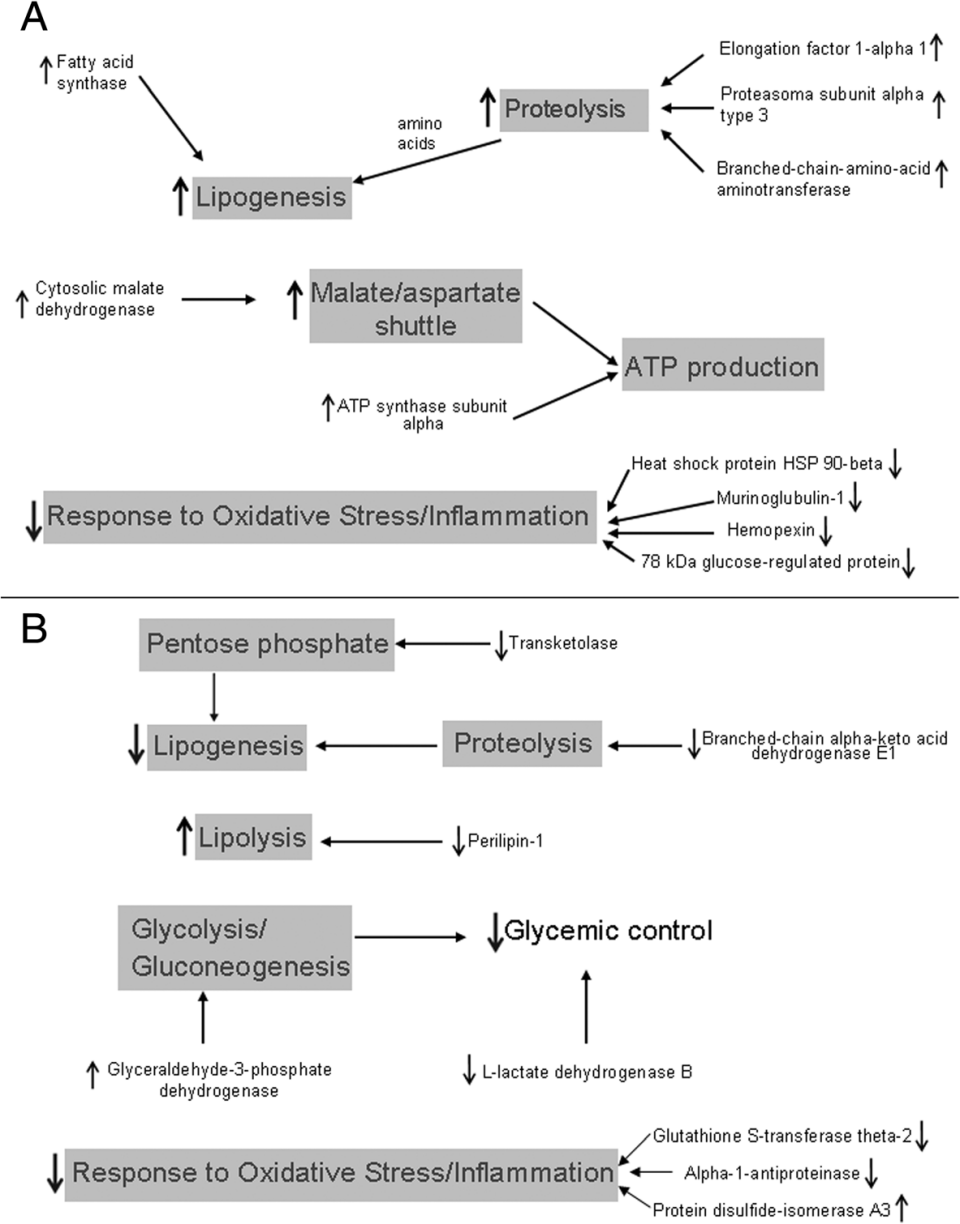
Diagram of the suggested pathways modified by IUGR in the retroperitoneal adipose tissue of male (**a**) and female (**b**) rats

In the restricted females, the decreased levels of perilipin-1 are indicative of increased lipolysis while the low levels of mitochondrial branched-chain alpha-keto acid dehydrogenase E1 indicate low proteolysis rate. The low levels of transketolase could represent low activity of the pentose phosphate pathway and, consequently, decreased lipogenesis rate. Down-regulation of L-lactate dehydrogenase may lead to impairment of glycemic control. These alterations point to a metabolic status of established obesity in the restricted females. In both genders, the protein variations indicated impairment of pathways involved in the responses to oxidative stress and inflammation (Fig. 5).

## Methods

### Rats

Wistar rats were cared for in accordance with the guidelines of the committee on animal research ethics of the Federal University of São Paulo (approval 486691). Three months-old rats were mated and the first day of pregnancy was determined by examination of vaginal smears for the presence of sperm. From day 1 of pregnancy, the dams were randomly assigned to be a control or a restricted dam. The control dams were fed ad libitum throughout pregnancy and lactation. The restricted dams received only 50 % of control intake during the whole pregnancy and were fed ad libitum during lactation. On the day of delivery, the pups were adjusted to eight per dam.

After weaning, the male and female offspring from control and restricted dams were housed four/five per cage and fed ad libitum until 4 months of age. The food provided to dams and offspring consisted of standard rat chow (Nuvital Nutrients, Columbo, PR, Brazil) containing (w/w) 4.5 % fat, 23 % protein, and 33 % carbohydrate, with 2.7 kcal/g, as determined at the Bromatology Division of the Federal University of São Paulo. All animals were maintained in controlled conditions of lighting (12-h light/12-h dark cycle, lights off at 18:00 h) and temperature (24 ± 1 °C) and had free access to water throughout the experimental period.

The numbers of animals used in the study were 16 male and 14 female controls and 17 male and 14 female restricted rats.

### Weight gain, weight of white adipose tissue and blood and tissue measurements

Food intake and body weight were measured once a week since weaning. At 4 months of age, the animals were killed by decapitation. The retroperitoneal adipose tissue was rapidly removed, weighed and frozen in liquid nitrogen. The tissue was stored at - 80 °C until analysis. The gonadal and mesenteric white adipose tissues were dissected and weighed.

Trunk blood was centrifuged and the serum stored at - 80 °C. Glucose analysis was performed by the glucose oxidase method, using a commercially available kit with detection limit of 0.32 mg/dL (Glucose Pap Liquiform, Labtest Diagnostica, São Paulo, Brazil). Triglycerides levels were determined using a commercially available kit with detection limit of 0.82 mg/dL (Labtest Diagnostica, São Paulo, Brazil). Insulin, corticosterone, adiponectin, TNF-α and IL-1β levels were measured by multiplex kit (Millipore, Bedford, MA, USA). The measurements of TNF-α, IL-10 and IL-6 in tissue were performed by Elisa (Millipore, Bedford, MA, USA).

### Proteome analysis

#### Sample preparation

An aliquot of 700 mg of retroperitoneal adipose tissue was homogenized in 1 ml of extraction buffer (7 M urea, 2 M thiourea, 4 % (w/v) CHAPS, 0.5 % (v/v) Triton X-100) containing complete Mini Protease Inhibitor Cocktail Tablets (Roche Diagnostics, Germany), added immediately before use. Sample lysates were centrifuged (19,000 g/30 min.) and supernatants stored at - 80 °C until analysis.

#### Protein assay

Protein concentration of supernatants was determined using 2-D Quant Kit (GE Healthcare, Pittsburgh, USA) and bovine albumin as standard, according to manufacturer’s recommendations.

#### Protein precipitation

Aliquots of 900 μg of protein were precipitated with a solution of 35 % KCl, 44 % chloroform, and 21 % methanol (v/v). The mixture was homogenized and centrifuged at 19,000 g and 4 °C for 15 min. The pellet was air-dried at room temperature.

#### Two-dimensional gel electrophoresis and image analysis

For isoelectric focusing (IEF), the pellet was dissolved in 500 μL of rehydration buffer (7 M urea, 2 M thiourea, 4 % (w/v) CHAPS, 0.5 % (v/v) Triton X-100, 100 mM DTT, 0.2 % (v/v) IPG Buffer pH 3–10, and traces of Bromofenol blue). IEF was carried out on a Protean IEF cell (Bio-Rad, CA, USA) using immobiline dry strips (18 cm linear gradient, pH 3–10) previously rehydrated for 12–14 h. IEF was performed with the current limit set at 50 mA per IPG strip with the following conditions at 18 °C: 100 V for 30 min, 250 V for 2 h, 500 V for 30 min, 1000 V for 30 min, 2000 V for 30 min, 4000 V for 1 h, 8000 V for 1 h followed by 8000 V until 30000 Vh.

After focusing, strips were equilibrated for 25 min in buffer containing 6 M urea, 50 mM Trisma base pH 8.8, 34 % (v/v) glycerol, 2 % (w/v) SDS, and 1 % (w/v) DDT, followed by an additional 25 min in the same buffer containing 2.5 % (w/v) iodoacetamide instead of DTT. Strips were then loaded onto 12 % SDS- polyacrylamide gels. After running in Protean II Multi-Cell (Bio-Rad, CA, USA), at 50 mA per gel for 6 h, the gels were stained for 48 h with Coomassie Blue G-250 (Bio-Rad, CA, USA). Stained gels were scanned (GS-710 Calibrated Imaging Densitometer) and analyzed using PDQuest Image Analysis Software version 7.2 (Bio-Rad, CA, USA).

#### Matrix-assisted laser desorption ionization time-of-flight mass spectrometry

The selected spots were manually excised, distained and digested. The spots were excised and distained in 50 % methanol and 5 % acetic acid overnight. The excised spots were treated with 25 mM ammonium bicarbonate and 50 % acetonitrile (1:1) and dried in SpeedVac. To the dried spots, 10 mM DTT was added and incubated for 1 h at 56 °C, followed by 55 mM IAA for 45 min on the dark. The spots were dehydrated with 25 mM ammonium bicarbonate followed by 25 mM ammonium bicarbonate with 50 % acetonitrile and dried in SpeedVac. Digestion was performed overnight with 15 ng of trypsin (Promega, WI, USA) in 25 mM ammonium bicarbonate, at 37 ° C. Digested samples were desalted using C18 Zip Tips (Millipore, Bedford, MA, USA). Two microliters of sample were applied on the spectrometer plate and air-dried at room temperature. The matrix solution (10 mg/mL α-cyano-4 hydroxycinnamic acid in 70 % acetonitrile/0.1 % trifluoroacetic acid) was applied on the spectrometer plate and air-dried at room temperature.

MALDI-TOF/TOF MS was performed using an Axima Performance ToF-ToF, (Kratos-Shimadzu Biotech, Manchester, UK) mass spectrometer. The instrument was externally calibrated with [M + H]^+^ ions of bradykinin (1–7 fragment, 757.4 Da), human angiotensin II (1046.54 Da), P14R synthetic peptide (1533.86 Da), and human ACTH (18–39 fragment, 2465.20 Da). Following MALDI MS analysis, MALDI MS/MS was performed on the 7 most abundant ions from each spot.

MASCOT (Matrix Science, UK) server was used to search Swiss-Prot protein database (http://www.matrixscience.com). The following parameters were used in this search: no restrictions on protein molecular weight, trypsin digest with one missing cleavage, monoisotopic mass, taxonomy limited to Rattus, carbamidomethylation of cysteine as fixed modification, possible oxidation of methionine and tryptophan, peptide mass tolerance of 0.5 Da, fragment mass tolerance of 0.8 Da, and peptide charge +1. False discovery rate (FDR) assessment was estimated using Mascot decoy database approach and only proteins identified with 0 % FDR were included in the results. Protein matching probabilities were determined using MASCOT protein scores, with identification confidence indicated by the number of matching and the coverage of protein sequence by the matching peptides. The presence of at least one peptide with significant ion score was required for positive protein identification. Only statistically significant MASCOT score results (*p* < 0.05) were included in the analysis.

The identified proteins were classified in Panther (http://www.pantherdb.org/) according to biological process.

#### Western Blot analysis

A sub-set of adipose tissue samples was used in western blot experiments. A 700 mg aliquot was homogenized in 1.0 ml of solubilization buffer (10 mM EDTA, 100 mM Tris pH 7.5, 10 mM sodium pyrophosphate, 100 mM sodium fluoride, 10 mM sodium orthovanadate, 2 mM PMSF, aprotinin 2 μg/mL, and 1 % Triton X-100). Insoluble material was removed by centrifugation (19,000 g at 4 ° C for 40 min.). The supernatant was collected and one aliquot was separated for protein concentration determination. Tissue extracts were denatured by boiling for 5 min in Laemmli buffer [90] containing 100 mM DTT. The protein concentration was determined by colorimetric method (BCA Protein Assay, Bioagency Biotecnologia, Brazil).

Subsequently, protein extracts (100 μg) were resolved in 12 % SDS polyacrylamide gels and transferred to nitrocellulose membranes using a semi-dry transfer system (Bio-Rad, CA, USA). Non-specific binding sites were blocked for 2 h in 1 % bovine serum albumin. The nitrocellulose membranes were then incubated overnight with primary antibody and for 1 h with the appropriate secondary antibody conjugated with horseradish peroxidase. The quantitative analysis was performed by densitometry using Scion Image software (Scion Corporation, Frederick, MD, USA).

The results were expressed in arbitrary units, as percentage changes in relation to the control group. For evaluation of protein loading, all membranes were stripped and reblotted with anti-β-tubulin (for male) and anti-β-actin (for female) primary antibody. The antibodies against 78 kDa glucose regulated protein (1:1000; ab53068), mitochondrial stress 70 protein (1:1000; ab106654), and glutathione S-transferase theta-2 (1:2500; ab102045) were obtained from ABCAM (Cambridge, UK). The antibody against β-tubulin (1:5000; #2146S) was purchased from Cell Signaling (Danvers, MA, USA). The antibody against β-actin (1:1000; sc-130657) was purchased from Santa Cruz (Dallas, TX, USA).

### Statistical analysis

The data are expressed as mean ± SEM. Comparisons between groups (control and restricted) were performed by Student *t* test. Statistical significance was set at *p* < 0.05.

## Additional files

Additional file 1: Table S1. Optic density of the spots with significant differences between the control and the restricted groups. (XLSX 12 kb) Additional file 2: Table S2. Protein name, gene name and biological process of the proteins identified in the adipose tissue of males. (XLSX 11 kb) Additional file 3: Table S3. Protein name, gene name and biological process of the proteins identified in the adipose tissue of females. (XLSX 10 kb)

## Abbreviation

2DE: two-dimensional gel electrophoresis
ATP: adenosine triphosphate
BW: body weight
FC: Fold change
IL: interleukin
IUGR: intrauterine growth restriction
MW: Molecular weight
NADH: Nicotinamide adenine dinucleotide
TCA: trycarboxilic acid cycle
TNF-α: tumor nuclear factor alpha

## References

[1] Langley-Evans SC (2009). Nutritional programming of disease: unravelling the mechanism. J Anat.

[2] Breton C (2013). The hypothalamus-adipose axis is a key target of developmental programming by maternal nutrition manipulation. J Endocrinol.

[3] Hajj NE, Schneider E, Lehnen H, Haaf T (2014). Epigenetics and life-long consequences of an adverse nutritional and diabetic intrauterine environment. Reproduction.

[4] Aiken CE, Ozanne SE (2013). Sex differences in developmental programming models. Reproduction.

[5] Picó C, Palou M, Priego T, Sánchez J, Palou A (2012). Metabolic programming of obesity by energy restriction during the perinatal period: different outcomes depending on gender and period, type and severity of restriction. Front Physiol.

[6] Fisher RE, Steele M, Karrow NA (2012). Fetal programming of the neuroendocrine-immune system and metabolic disease. J Preg.

[7] Howie GJ, Sloboda DM, Vickers MH (2012). Maternal undernutrition during critical windows of development results in differential and sex-specific effects on postnatal adiposity and related metabolic profiles in adult rat offspring. Br J Nutr.

[8] Manuel-Apolinar L, Rocha L, Damasio L, Tesoro-Cruz E, Zarate A (2014). Role of prenatal undernutrition in the expression of serotonin, dopamine and leptin receptor in adult mice: implication of food intake. Mol Med Reports.

[9] Anguita RM, Sigulem DM, Sawaya AL (1993). Intrauterine food restriction is associated with obesity in young rats. J Nutr.

[10] Porto LCJ, Sardinha FLC, Telles MM, Guimarães RB, Albuquerque KT, Andrade IS, Oyama LM, Nascimento CMO, Santos OFP, Ribeiro EB (2009). Impairment of the serotonergic control of feeding in adults female rats expose to intra-uterine malnutrition. Br J Nutr.

[11] Sardinha FLC, Telles MM, Albuquerque KT, Oyama LM, Guimarães PAMP, Santos OFP, Ribeiro EB (2006). Gender difference in the effect of intrauterine malnutrition on the central anorexigenic action of insulin in adult rats. Nutrition.

[12] Vickers MH, Breier BH, Cutfield WS, Hofman PL, Gluckman PD (2000). Fetal origins of hyperphagia, obesity, and hypertension and postnatal amplification by hypercaloric nutrition. Am J Physiol.

[13] Bieswal F, Ahn M, Reusens B, Holvoet P, Raes M, Rees WD, Remacle C (2006). The importance of catch-up growth after early malnutrition for the programming of obesity in male rat. Obesity.

[14] Delahaye F, Lukaszewski M-A, Wattez J-S, Cisse O, Dutriez-Casteloot I, Fajardy I, Montel V, Dickes-Coopman A, Laborie C, Lesage J, Breton C, Vieau D (2010). Maternal perinatal undernutrition programs a “brown-like” phenotype of gonadal white fat in male rat at weaning. Am J Physiol Regul Integr Comp Physiol.

[15] Alexandre-Goubau M-CF, Courant F, Le-Gall G, Moyon T, Darmaun D, Parnet P, Coupé B, Antignac J-P (2011). Offspring metabolomic response to maternal protein restriction in a rat model of intrauterine growth restriction (IUGR). J Proteome Res.

[16] Proença ARG, Sertié RAL, Oliveira AC, Campaña AB, Caminhotto RO, Chimin P, Lima FB (2014). New concepts in White adipose tissue physiology. Braz J Med Biol Res.

[17] Belfiore F, Rabuazzo AM, Napoli E, Borzi V, Vecchio LL (1975). Enzymes of glucose metabolism and of citrate cleavage pathway in adipose tissue of normal and diabetes subjects. Diabetes.

[18] Langin D (2006). Adipose tissue lipolysis as a metabolic pathway to define pharmacological strategies against obesity and metabolic syndrome. Pharmacol Res.

[19] Dessì A, Pravettoni C, Marincola FC, Schirru A, Fanos V (2015). The biomarkers of fetal growth in intrauterine growth retardation and large for gestational age cases: from adipocytokines to a metabolomic all-in-one tool. Expert Rev Proteomics.

[20] Kershaw EE, Flier JS (2004). Adipose tissue as an endocrine organ. J Clin Endocrinol Metab.

[21] Nascimento CMO, Ribeiro EB, Oyama LM (2009). Metabolism and secretory function of white adipose tissue: effect of dietary fat. Anais Acad Bras Cienc.

[22] Briana DD, Malamitsi-Puchner A (2009). Intrauterine growth restriction and adult disease: the role of adipocytokines. Eur J Endocrinol.

[23] Ibáñez L, Sebastiani G, Lopez-Bermejo A, Díaz M, Gómez-Roig MD, de Zegher F (2008). Gender specificity of body adiposity and circulating adiponectin, visfatin, insulin, and insulin growth factor-I at term birth: relation to prenatal growth. J Clin Endocrinol Metab.

[24] Fuchs D, Winkelmann I, Johnson IT, Mariman E, Wenzel U, Daniel H (2005). Proteomics in nutrition research: principles, technologies and applications. Br J Nutr.

[25] Wang J, Li D, Dangott LJ, Wu G (2006). Proteomics and its role in nutrition research. J Nutr.

[26] Roepstorff P (2012). Mass spectrometry-based proteomics, background, status and future needs. Protein Cell.

[27] Silva TS, Richard N, Dias JP, Rodrigues PM (2014). Data visualization and futures selection methods in gel-based proteomics. Cur Protein Pep Sci.

[28] You Y-A, Lee JH, Kwon EJ, Yoo JY, Kwon W-S, Pang M-G, Kim YJ. Proteomic analysis of one-carbon metabolism-related marker in liver of rat offspring. Mol Cel Proteomics. 2015. Paper in press. 10.1074/mcp.M114.046888 PMC4638034 26342040

[29] Sarr O, Louveau I, Kalbe C, Metges CC, Rehfeldt C, Gondret F (2010). Prenatal exposure to maternal low or high protein diets induces modest changes in the adipose tissue proteome of newborn piglets. J Anim Sci.

[30] Ruis-Gonzáles MD, Cañete MD, Gómez-Chaparro JL, Abril N, Cañete R, López-Barea J (2015). Alteration of protein expression in serum of infants with intrauterine growth restriction and different gestational age. J Proteomics.

[31] Alexandre-Goubau M-CF, Bailly E, Moyon TL, Grit IC, Coupé B, Drean GL, Rogniaux HJ, Parnet P (2012). Postnatal growth velocity modulates alterations of proteins involved in metabolism and neuronal plasticity in neonatal hypothalamus in rats born with intrauterine growth restriction. J Nutr Biochem.

[32] Fabricius-Bjerre S, Jensen RB, Faerch K, Larsen T, Molgaard C, Michaelsen KF, Vaag A, Greisen G (2011). Impact of birth weight and early infant weight gain on insulin resistance and associated cardiovascular risk factors in adolescence. Plos One.

[33] Ravelli ACJ, van der Meuelen JHP, Osmond C, Barker DJP, Bleker OP (1999). Obesity at the age 50 y in men and women exposed to famine prenatally. Am J Clin Nutr.

[34] Desai M, Han G, Ferelli M, Kallichanda N, Lane RH (2008). Programmed upregulation of adipogenic transcriptions factors in intrauterine growth-restricted offspring. Reprod Sci.

[35] Lukaszewski M-A, Mayer S, Fajardy I, Delahaye F, Dutriez-Casteloot I, Montel V, Dickes-Coopman A, Laborie C, Lesage J, Vieau D, Breton C (2011). Maternal prenatal undernutrition programs adipose tissue gene expression in adult male rat offspring under high-fat diet. Am J Physiol Endocrinol Metab.

[36] Berndt J, Kovacs P, Ruschke K, Klöting N, Fasshauer M, Schön MR, Körner A, Stumvoll M, Blüher M (2007). Fatty acid synthase gene expression in humana adipose tissue: assossiation with obesity and type 2 diabetes. Diabetologia.

[37] Vessal M, Mishra S, Moulik S, Murphy LJ (2006). Prohibitin attenuates insulin-stimulated glucose and fatty acid oxidation in adipose tissue by inhibition of pyruvate carboxylase. FEBS J.

[38] Ande SR, Nguyen KH, Padilla-Meier GP, Wahida W, Nyomba BLG, Mishra S (2014). Prohibitin overexpression in adipocytes induces mitochondrial biogenesis, leads to obesity development, and affects glucose homeostasis in a sex-specific manner. Diabetes.

[39] Liu D, Lin Y, Kang T, Huang B, Xu W, Garcia-Barro M, Olatinwo M, Matthews R, Chen YE, Thompson WE (2012). Miochondrial dysfunction and adipogenic reduction by prohibitin silencing in 3 T3-L1 cells. PLoS One.

[40] Herman MA, She P, Peroni OD, Lynch CJ, Kahn BB (2010). Adipose tissue branched chain amino acid (BCAA) metabolism modulates circulating BCAA levels. J Biol Chem.

[41] Kowalski TJ, Wu G, Watford M (1997). Rat adipose tissue amino acid metabolism in vivo as assessed by microdialysis and arteriovenous techniques. Am J Physiol.

[42] Patterson BW, Horowitz JF, Wu G, Watford M, Coppack SW, Klein S (2011). Regional muscle and adipose tissue amino acid metabolism in lean and obese women. Am J Physiol Endocrinol Metab.

[43] Badoud F, Lam KP, DiBattista A, Perreault M, Zulyniak MA, Cattrysse B, Stephenson S, Britz-McKibbin P, Mutch DM (2014). Serum and adipose tissue amino acid homeostasis in the metabolically healthy obese. J Proteome Res.

[44] Hershey JW (1991). Translational control in mammalian cell. Annu Rev Biochem.

[45] Thornton S, Anand N, Purcell D, Lee J (2003). Not just for housekeeping: protein initiation and elongation factors in cell growth and tumorigenesis. J Mol Med.

[46] Chuang S-M, Chen L, Lambertson D, Anand M, Kinzy TG, Madura K (2005). Proteasome-mediated degradation of cotranslationally damage proteins involves translation elongation factor 1A. Mol Cell Biol.

[47] Al-Maghrebi M, Anin JT, Olalu AA (2005). Up-regulation of eukaryotic elongation factor 1 subunits in breast carcinoma. Anticancer Res.

[48] Al-Maghrebi M, Cojocel C, Thompson MS (2005). Regulation of elengation factor 1 expression by vitamin E in diabetic rat kidney. Mol Cell Biochem.

[49] Hanzu FA, Vinaixa M, Papageourgiou A, Párrizas M, Correig X, Delgado S, Carmona F, Samino S, Vidal J, Gomis R (2014). Obesity rather than regional fat depots marks the metabolomic pattern of adipose tissue: an untargeted metabolomic approach. Obesity.

[50] Favretto D, Cosmi E, Ragazzi E, Visentin S, Tucci M, Fais P, Cecchetto G, Zanardo V, Viel G, Ferrara SD (2012). Cord blood metabolomic profiling in intrauterine growth restriction. Anal Bioanal Chem.

[51] Lee S-M, Dho SH, Ju S-K, Maeng J-S, Kim J-Y, Kwon K-S (2012). Cytosolic malate dehydrogenate regulates senescence in human fybroblasts. Biogereontology.

[52] Dessì A, Puddu M, Ottonello G, Fanos V (2013). Metabolomics and fetal-neonatal nutrition: between “not enough” and “too much”. Molecules.

[53] Mali Y, Zisapels N (2008). Gain of interaction of ALS-linked G93A superoxide dismutase with cytosolic malate dehydrogenase. Neurobiol Dis.

[54] Minárik P, Tomásková N, Kollárová M, Antalík M (2002). Malate dehydrogenase – structure and function. Gen Physiol Biophys.

[55] Theys N, Ahn M-T, Bouckenooghe T, Reusens B, Remacle C (2011). Maternal malnutrition programs pancreatic islet mitochondrial dysfunction in the adult offspring. J Nutr Biochem.

[56] Saito A, Shinohara H (1985). Rat plasma murinoglobulin: isolation, characterization, and comparison with rat α-1- and α-2-macroglobulins. J Biochem.

[57] Tolosano E, Altruda F (2002). Hemopexin: structure, function, and regulation. DNA Cell Biol.

[58] Jung JY, Kwak YH, Kim KS, Kwon WY, Suh GJ (2015). Change of hemopexin level is associated with the severity of sepsis in endotoxemic rat model and the outcome of septic patients. J Crit Care.

[59] Gianazza E, Sensi C, Eberini I, Gilardi F, Giudici M, Crestani M (2013). Inflammatory serum proteome pattern in mice fed a high-fat diet. Amino Acids.

[60] Walter P, Ron D (2011). The unfolded protein response: to stress pathway to homeostatic regulation. Science.

[61] Özcan U, Cao Q, Yilmaz E, Lee A-H, Iwakoshi NN, Özdelen E, Tuncman G, Görgün C, Glimcher LH, Hotamisligil GS (2004). Endoplasmic reticulum stress links obesity, insulin action, and type 2 diabetes. Science.

[62] Teodoro-Morrison T, Schuiki I, Zhang L, Belsham DD, Volchuk A (2013). GRP78 overproduction in pancreatic beta cells protects against high-fat-diet-induced diabetes in mice. Diabetologia.

[63] Lanneau D, Brunet M, Frisan E, Solary E, Fontenay M, Garrido C (2008). Heat shock proteins : essential proteins for apoptosis regulation. J Cell Mol Med.

[64] Tiss A, Khadir A, Abubaker J, Abu-Farha M, Al-Khairi I, Cherian P, John J, Kavalakatt S, Warsame S, Al-Ghimlas F, Elkum N, Behbehani K, Dermime S, Dehbi M (2014). Immunohistochemical profiling of the heat shock response in obese non-diabetic subjects revealed impairment expression of heat shock proteins in the adipose tissue. Lipids Health Dis.

[65] Saggerson ED, McAllister TWJ, Bath HS (1988). Lipogenesis in rat brown adipocytes – effects of insulin and noradrenaline, contributions from glucose and lactate as precursors and comparisons with white adipose tissue. Biocem J.

[66] O'Hea EK, Leveille G (1969). Significance of adipose tissue and liver as sites of fatty acid synthesis in the pig and the efficiency of utilization of various substrates for lipogenesis. J Nutr.

[67] van Hall G (2010). Lactate kinects in human tissues at rest and during exercise. Acta Physiol.

[68] Sabbater D, Arriarán S, Romero MM, Agnelli S, Remesar X, Fernández-López JA, Alemany M (2014). Cultured 3 T3-L1 adipocytes dispose of excess medium glucose as lactate under abundant oxygen availability. Sci Rep.

[69] Arriaran S, Agnelli S, Sabater D, Remesar X, Fernádez-López JA, Alemany M (2015). Evidences of basal lactate production in the main white adipose tissue sites of rats. Effect of sex and a cafeteria diet. PLoS One.

[70] Dugail I, Quignard-Boulange A, Bazin R, Le Liepvre X (1988). Adipo-tissue-specific increase in glyceraldehyde-3-phosphate dehydrogenase activity and mRNA amounts in suckling pre-obese Zucker rats. Biochem J.

[71] Rattanatray L, MacLaughlin SM, Kleemann DO, Walker SK, Muhlhausler BS, McMillen IC (2010). Impact of maternal periconceptional overnutrion on fat mass and expression of adipogenic and lipogenic genes in visceral and subcutaneous fat depots in the postnatal lamb. Endocrinology.

[72] Pérez-Pérez R, García-Santos E, Ortega-Delgado FJ, López JA, Camafeita E, Ricart W, Fernández-Real J-M, Peral B (2012). Attenuated metabolism is a hallmark of obesity as revealed by comparative proteomic analysis of human omental adipose tissue. J Proteomics.

[73] Brasaemle DL (2007). The perilipin family of structural lipid droplet proteins: stabilization of lipid droplets and control of lipolysis. J Lipid Res.

[74] Ray H, Pinteur C, Frering V, Beylot M, Large V. Depot-specific differences in perilipin and hormone-sensitive lipase expression in lean and obese. Lipid Health Dis. 2009;8(58). doi:10.1186/1476-511X-8-58. 10.1186/1476-511X-8-58 PMC2804660 20017959

[75] Tansey J, Sztalryd C, Gruia-Gray J, Roush DL, Zee JV, Gavrilova O, Reitman ML, Deng CX, Li C, Kimmel AR, Londos C (2001). Perilipin ablation results in a lean mouse with aberrant adipocyte lipolysis, enhanced leptin production, and esistance to diet-induced obesity. Proc Natl Acad Sci.

[76] Ballatory N, Krance SM, Notenboom S, Shi S, Tieu K, Hammond CL (2009). Glutathione dysregulation and the etiology and progression of human disease. Biol Chem.

[77] Kharb S (2000). Low whole blood glutathione levels in pregnancies complicated by preeclampsia and diabetes. Clin Chim Acta.

[78] Boden G, Duan X, Homko C, Molina EJ, Song W, Perez O, Cheung P, Merali S (2008). Increase in endoplasmic reticulum stress-related proteins and gene in adipose tissue of obese, insulin-resistant individuals. Diabetes.

[79] Fuentes-Almagro CA, Prieto-Álamo M-J, Pueyo C, Jurado J (2012). Identification of proteins containing redox-sensitive thiols after PRDX1, PRDX3 and GCLC silencing and/or glucose oxidase treatment in Hepa 1–6 cells. J Prot.

[80] Nonn L, Berggren M, Powis G (2003). Increased expression of mitochondrial peroxiredoxin-3 (thioredoxin peroxidase-2) protects cancer cells against hypoxia and drug-induced hydrogen peroxide-dependent apoptosis. Mol Cancer Res.

[81] Chang T-S, Cho C-S, Park S, Yu S, Kang SW, Rhee SG (2004). Peroxiredoxin III, a mitochondrion-specific peroxidase, regulates apoptotic signaling by mitochondria. J Biol Chemis.

[82] Janciauskiene S, Larsson S, Larsson P, Virtala R, Jansson L, Stevens T (2004). Inhibition of lipopolysaccharide-mediated human monocyte activation, in vitro, by α1-antitrypsin. Biochem Biophys Res Comm.

[83] Stockley RA, Shaw J, Afford SC, Morrison HM, Burnett D (1990). Effect of alpha-1-proteinase inhibitor on neutrophil chemotaxis. Am J Respir Cell Mol Biol.

[84] Bergin DA, Reeves EP, Meleady P, Henry M, McElvaney OJ, Carroll TP, Condron C, Chotirmall SH, Clynes M, O'Neill SJ, McElvaney NG (2010). α-1 Antitrypsin regulates human neutrophil chemotaxis induced by soluble immune complexes and IL-8. J Clin Invest.

[85] Al-Omari M, Korenbaum E, Ballmaier M, Lehmann U, Jonigk D, Manstein DJ, Welte T, Mahadeva R, Janciauskiene S (2011). Acute-phase protein α1-antitrypsin inhibits neutrophil calpain I and induces random migration. Mol Med.

[86] Griese M, Latzin P, Kappler M, Weckerle K, Heinzlmaier T, Bernhardt T, Hartl D (2007). α1-antitripsin inhalation reduces airway inflammation in cystic fibrosis patients. Eur Respir J.

[87] Kalis M, Kumar R, Janciauskiene S, Salehi A, Cílio CM (2010). α1-antitripsinenhances insulin secretion and prevents cytokine-mediated apoptosis in pancreatic β-cells. Islet.

[88] Lu Y, Tang M, Wasserfall C, Kou Z, Campbell-Thompson M, Gardemann T, Crawford J, Atkinson M, Song S (2006). α1-antitrypsin gene therapy modulates cellular immunity and efficiently prevents type 1 diabetes in nonobese diabetic mice. Human Gene Terapy.

[89] Oliva K, Barker G, Rice GE, Bailey MJ, Lappas M (2013). 2D-DIGE to identify proteins associated with gestational diabetes in omental adipose tissue. J Endocrinol.

[90] Laemmli UK (1970). Cleavage of structural protein during the assembly of the head of bacteriophage T4. Nature.

